
JOURNAL INFORMATION
==============================
NLM Title Abbreviation: medRxiv
NLM Journal ID: 101767986
Journal ID: MEDRXIV

medRxiv

Publisher: Cold Spring Harbor Laboratory Preprints

ARTICLE INFORMATION
==============================
PMCID: PMC12622079
PMID: 41256145
DOI: 10.1101/2025.09.29.25335807
Article version: 2
Article version: preprint
Article version: 2
Subjects: Article

Longterm Impact of COVID-19 in Hospitalized and Ambulatory Jamaican Children

http://orcid.org/0009-0008-6052-4259 Mosha Miller Samantha Ann-Patrice BA, BSc, MBBS
http://orcid.org/0000-0002-1768-5419 Hicar Mark Daniel MD, PhD
http://orcid.org/0000-0002-9149-889X Berry Crista-Lee Shahine MBBS, DM Peds, RCPSC-SEAP(Resp)
http://orcid.org/0000-0002-5725-2351 Anderson Kathryn B. MD, PhD, CTrop Med
http://orcid.org/0000-0002-4781-4998 Lindo John F. PhD
http://orcid.org/0000-0001-6758-0941 Morse Gene D. PharmD
http://orcid.org/0000-0002-8981-3934 Christie Celia Dana Claire MBBS, DM Peds, MPH *
Co-authors:

* Corresponding author: Celia.ChristieSamuels@uwimona.edu.jm
Electronic publication date: 2025 Oct 21
Electronic Location ID: 2025.09.29.25335807
License: This work is licensed under a Creative Commons Attribution 4.0 International License, which allows reusers to distribute, remix, adapt, and build upon the material in any medium or format, so long as attribution is given to the creator. The license allows for commercial use.
License URL: https://creativecommons.org/licenses/by/4.0/

nihpp-2025.09.29.25335807.pdf
Long COVID-19 in Hospitalized and Ambulatory Children in Jamaica 45 10 11 5 2026 885 887 The Pediatric Infectious Disease Journal 10.1097/INF.0000000000005259 PMC13557477 42108528

==============================
Withdrawal statement:

The authors have withdrawn their manuscript because it was posted without the consent of all individuals listed in the acknowledgements. Therefore, the authors do not wish this work to be cited as reference for the project. If you have any questions, please refer to the resubmission of this article, or please contact the corresponding author.

